# Building Capacity and Infrastructure at Hospitals Implementing Minimally Invasive Tissue Sampling: Experience and Lessons Learned From Nepal, Rwanda, and Tanzania

**DOI:** 10.1093/cid/ciab780

**Published:** 2021-12-15

**Authors:** Nuwadatta Subedi, Suraj Bhattarai, Alex Mremi, Gervais Ntakirutimana, Marie Claire Ndayisaba, Belson Rugwizangoga, Djibril Mbarushimana, Elisée Hategekimana, Vestine Tuyizere, Christina Paganelli

**Affiliations:** 1 Gandaki Medical College Teaching Hospital and Research Center, Gandaki Province, Pokhara, Nepal; 2 Global Institute for Interdisciplinary Studies, Kathmandu, Nepal; 3 Department of Pathology, Kilimanjaro Christian Medical Centre and Kilimanjaro Christian Medical University College, Moshi, Tanzania; 4 (CHUK), Kigali, Rwanda; 5 Centre Hospitalier Universitaire de Butare (CHUB), Huye, Rwanda; 6 Research Triangle Institute (RTI) International, Seattle, Washington, USA

**Keywords:** autopsy, cause of death, low- and middle-income countries, minimally invasive tissue sampling, mortality surveillance

## Abstract

**Background:**

Minimally invasive tissue sampling (MITS) is a useful tool to determine cause of death in low- and middle-income countries (LMICs). In 2019 the MITS Surveillance Alliance supported the implementation of small-scale postmortem studies using MITS in several LMICs.

**Methods:**

In this article we describe the preparations, challenges, and lessons learned as part of implementing MITS across 4 study sites in 3 countries: Nepal, Rwanda, and Tanzania. We describe the process for building capacity to conduct MITS, which consisted of training in MITS sample collection, individual site assessment to determine readiness and gaps prior to implementation, site visits as sites began implementation of MITS, and feedback based on remote evaluation of histology slides via an online portal.

**Results:**

The 4 study sites each conducted 100 MITS, for a total of 400. All 4 sites lacked sufficient infrastructure and facilities to conduct MITS, and upgrades were required. Common challenges faced by sites included that clinical autopsies were neither routinely conducted nor widely accepted. Limited clinical records made cause of death determination more difficult. Lessons learned included the importance of sensitization of the community and medical staff to MITS to enhance understanding and increase consent.

**Conclusions:**

The study sites accomplished MITS and utilized the available support systems to overcome the challenges. The quality of the procedures was satisfactory and was facilitated through the organized capacity-building programs.

Minimally invasive tissue sampling (MITS) involves sampling of select organs and bodily fluids using biopsy needles for histological and microbiological analyses. MITS has been increasingly used as a possible alternative to complete diagnostic autopsy (CDA) to determine cause of death, especially in low- and middle-income countries (LMICs) [1, 2]. MITS can be an efficient tool for clinical autopsy cases, and results have shown high concordance with CDA [3–7]. MITS can be incorporated to support accurate cause of death determination, particularly for cases lacking a clinical diagnosis or deaths where there are discrepancies in the clinic and pathological diagnosis, namely in infectious diseases [8]. Where CDA has limited acceptance, MITS has been well received by healthcare providers, parents, and religious leaders because it is not disfiguring and is less time consuming, thus causing fewer delays for funerals [9, 10].

## INCENTIVE GRANT PROJECTS SUPPORTED BY THE MITS ALLIANCE

The MITS Surveillance Alliance was established in 2017 as a global multidisciplinary consortium of researchers to expand the use of pathology-based postmortem examination and specifically the use of MITS. In 2019 the MITS Alliance funded 12 grants to implement small-scale postmortem studies using MITS, all in LMICs.

In this article, we describe the capacity building, development of infrastructure, challenges faced, and lessons learned implementing MITS at 4 study sites in 3 countries: Nepal, Rwanda, and Tanzania.

## METHODS

The process for building capacity and preparing for MITS implementation was consistent across sites and included training, site assessment, site visits, and ongoing quality assurance (QA). The characteristics of each study site and study population are outlined in Table 1. The details of capacity building are described in more detail in the accompanying article, Building Global Capacity to Conduct Pathology-Based Postmortem Examination: Establishing a New Training Hub for Minimally Invasive Tissue Sampling [11].

**Table 1.** Study Sites and Populations

			Study Population			
Site	Study Site	Eligibility Criteria	Perinatal Deaths (<7 days)	Children (<18 years)	Adults (>18 years)	Total
Nepal	Gandaki Medical College (GMC), Pokhara Academy of Health Sciences (PAHS), and Damauli Hospital, Tanahun	Deceased, >18 years old with deaths from known or suspected natural diseases	0	0	100	100
CHUK, Rwanda	Centre Hospitalier Universitaire de Kigali (CHUK)	Deaths from any age group from any health facility within the CHUK catchment area	1	1	98	100
CHUB, Rwanda	Centre Hospitalier Universitaire de Butare (CHUB)	Hospital deaths > 27 days of age	0	15	85	100
Tanzania	Kilimanjaro Christian Medical Center (KCMC)	Deceased adults and pediatric patients >2 months of age who died within 72 hours of hospitalization	0	17	83	100

### MITS Training

The training program began with a 5-day in-person course focused on acquisition of skills related to MITS sample collection. The training included demonstration and skills practice in MITS sample collection, including variations in age-specific MITS sampling, and orientation to the components of the MITS kit and their use. In anticipation of cascading the training to other study staff, participants were given opportunities to practice teaching sample collection and giving feedback to fellow participants.

### Site Assessment

A component of the training included a review of the minimum facilities required to conduct MITS and preparing participants to assess their site prior to implementation. During the training each team drafted a sample schematic process for MITS operations in their own projects. After completing the training, participants returned to their site and conducted an assessment of site readiness including available human and material resources and capacity (Table 2). Site assessment consisted of identifying the minimum amount of space, necessary materials, and organization of the space to best support MITS implementation. The minimal facility requirements and equipment needed to perform MITS in hospital settings is presented in Table 2.

**Table 2.** Minimum Requirements for MITS Set-up and Functioning in LMIC Settings

Facilities and Infrastructure	Equipment for Sample Collection
• Refrigerated storage for bodies • Sample collection: •A well-ventilated and well-lit room proximate to hospital and restricted from public access •Autopsy table with a water supply and drainage •Clearly marked biohazard waste disposal •Secure storage room for MITS kits •Changing room •Washroom • Laboratory with histopathology and microbiology facilities including microscopes, reagents, tissue processing machine, microtome and embedding tools • Research office with computer, printer etc.	• Personal protective equipment including surgical cap, impermeable disposable gown, surgical gloves, mask, eye protection and shoe covers • Basic set of surgical instruments (scissors, tissue holding forceps, hemostats, surgical trays, etc.) • MITS kit with biopsy needles, syringe, test tubes, sample collection jars, measuring tape, etc. (provided by the MITS Alliance) • Consumables (disinfectants, cotton, gauze, reagents for hemostasis, eg. Monsel’s solution etc.) • Digital camera

Abbreviations: LMIC, low- and middle-income country; MITS, minimally invasive tissue sampling.

### Implementation Visit

Once the study sites had initiated enrollment and begun implementing MITS, a visit by technical advisors was planned. The visit was targeted to support standard implementation of MITS, troubleshoot possible challenges, and provide additional feedback on sample collection and processing.

### Quality Assurance and Technical Assistance

Ongoing technical assistance to support quality assurance of MITS samples was supported in a variety of ways. An online Quality Assurance Portal was developed through the MITS website to provide remote support to trainees in ongoing quality improvement of MITS sample collection and processing for histopathology. Sites were asked to upload histology images from their initial cases for each tissue type for review by expert pathologists at the US Centers for Disease Control and Prevention. The evaluation criteria consisted of whether a sufficient amount of target tissue was obtained, the presence or absence of tissue folds, quality of staining, and overall quality of the histology slides. Once a total of five cases were deemed satisfactory by the reviewers, sites systematically uploaded images from 10% of the remaining MITS cases. The MITS Alliance also conducted regular technical working group meetings where the participants engaged in discussions and case reviews within the technical areas of pathology, microbiology, social behavioral sciences, and cause of death determination.

The period of performance of the study was September 2019 to June 2021. The MITS procedure at each study site was conducted per the standard operating procedure established by the MITS Alliance, with adaptations based on the study population [12].

## ETHICS APPROVALS

Each study was reviewed by the respective ethics review committee of its institution. In Nepal, ethical clearance was obtained from Ethical Review Board of Nepal Health Research Council. Institutional Ethics Committee of University Teaching Hospital of Kigali (CHUK) and the Research and Ethics Committee of the University Teaching Hospital of Butare (CHUB) approved respective studies in Rwanda and the College Research Ethics Review Committee of the Kilimanjaro Christian Medical University College and National Institute of Medical Research for the Tanzania site. In all cases, the closest relatives of the deceased present at the hospitals and capable of making decisions on behalf of the family provided informed consent to participate in the research study.

## RESULTS

### MITS Training

A total of 8 people, 2 from each site, 1 of whom was required to be a pathologist, participated in an in-person training conducted by the MITS Alliance. Following the initial training, participants from each site trained project staff in MITS sample collection, resulting in an additional 62 participants including pathologists, microbiologists, lab technicians, and other healthcare workers. Table 3 describes those trained in MITS sample collection at each site.

**Table 3.** Background of Participants Attending Trainings on the MITS Method

		Additional Project Staff Trained			
Site	Initial Training Participants	Pathologists	Microbiologists	Lab Technicians	Other Healthcare Workers
Nepal	2 forensic pathologists	1	1	4	3
CHUK Rwanda	1 pathologist and 1 histotechnologist	4	3	4	3
CHUB, Rwanda	1 pathologist and 1 nurse	…	…	15	15
Tanzania	1 anatomical pathologist, 1 junior medical officer	3	1	3	2

Abbreviations: CHUB, University Teaching Hospital of Butare; CHUK, University Teaching Hospital of Kigali; MITS, minimally invasive tissue sampling.

### Site Assessment

After establishing minimum standards for conducting MITS (Table 2), gaps in infrastructure and capacity were identified as part of the individual site assessment. Each site adapted existing facilities and infrastructure to facilitate the implementation of MITS consistent with the standards established as part of the MITS training. The facilities to perform autopsies were not adequate to permit the performance of MITS in a standard way, and upgrades and modifications were identified across all study sites.

### Implementation Visit

Three of the 4 sites, CHUB, CHUK, and Nepal, participated in implementation visits by technical advisors. The visits were 4 days in duration and were planned to take place after the site had initiated enrollment and had completed a minimum of 4 MITS cases. Because the Tanzania site had previously conducted a study involving MITS, an implementation visit was deemed unnecessary. Over the course of the visit technical advisors and site staff discussed the results of the site assessment conducted by the study staff reviewing the site infrastructure and adaptations, study logistics, staff capacity, and resources and project administration. Technical advisors also observed and provided guidance in MITS sample collection and processing and provided orientation to the QA telepathology system.

### Quality Assurance and Technical Assistance

The histology slides were uploaded to the QA portal of the MITS Alliance website according to the guidelines. In total, 80% of the initial cases submitted by all sites were rated as “satisfactory,” and nearly 90% of the remaining cases submitted were deemed “satisfactory.” All sites found the feedback about sample collection and processing in the initial cases useful for optimizing and improving techniques in subsequent cases.

## Discussion

The introduction of any intervention to determine cause of death could be challenging in a new setting where such procedures are not routinely performed. Capacity-development plans have proven to be successful in the standardization of MITS and histology procedures [13]. Many experiences implementing MITS from the 4 sites described in this article, including the challenges and lessons learned, were similar. The 4 sites agreed that MITS could be performed to support cause of death determination if capacity is adequate, proper protocols are followed, there is sufficient infrastructure, and there is stakeholder engagement.

### The Challenges of MITS Implementation at the Study Sites

In all sites postmortem examinations faced some challenges because most autopsies are performed for medicolegal requirements, and clinical autopsies are neither frequently performed nor widely accepted. Across sites family members and relatives of the deceased were prepared for cultural disposal of the body soon after death and at times were reluctant to consent to MITS for fear of delaying the funeral. Thus, the consenting process was perceived as one of the biggest challenges in implementation of MITS across the study sites. Additionally, sites experienced hesitancy to accept MITS by health care providers. At CHUB the responsibility for obtaining consent was given primarily to the bedside nurse or the treating general practitioner or resident. Although trained in providing counseling and consent for MITS, some nurses were not comfortable enough to engage in counseling during their duty and delegated counseling to others. In Nepal, the clinicians were initially concerned that MITS findings (diagnosis) might question the credibility of the clinical services provided.

All 4 sites initially faced deficits in terms of the infrastructure needed for conducting MITS. All sites upgraded mortuary facilities to either create or modify an existing autopsy room, designated to MITS. Two sites built new autopsy rooms, whereas 2 sites modified existing rooms by adding new doorways and improving light and ventilation. Sites also identified additional spaces for conducting MITS consent and a changing room for those performing MITS. In addition all sites either adapted existing autopsy tables or purchased new autopsy tables. At times, the lack of well-equipped laboratories and unavailability of some reagents at some sites limited the ability to analyze samples.

Human resources were limited, and all study sites were required to hire additional study staff based on their specific needs. In Nepal, 4 lab technicians were additionally trained to process the specimens obtained by MITS and engaged on a per case basis. Likewise, 2 assistants were hired to facilitate research. The existing manpower of the mortuaries, administrative staff, nurses, and resident doctors were engaged in the research as needed. Two additional part-time research assistants were also employed in Tanzania.

Lack of adequate clinical records of the deceased was a constraint in ascertaining cause of death at all sites. The unavailability was attributed to lack of digital medical record-keeping systems in Nepal and Rwanda. Reliable clinical information was the most difficult to obtain in cases that were not admitted to hospitals or were admitted for a very short period prior to death.

Once the study projects were underway, the coronavirus disease 2019 (COVID-19) pandemic affected the enrollment of cases throughout all 4 sites. Many governments imposed lockdowns that prevented nonessential work and imposed strict travel restrictions, making all aspects of project operations including enrollment and visiting other study sites difficult. In Nepal, MITS was complicated by COVID-19 because the criteria for polymerase chain reaction testing for COVID-19 in the deceased was unclear; in general, there was a decreased focus on postmortem examination including in COVID-19 cases. The initial absence of reliable guidelines for infection control contributed to generalized anxiety and fear of infection among study teams, resulting in hesitancy in carrying out field activities. Lab personnel were also reluctant to process samples collected from cases with unknown severe acute respiratory syndrome coronavirus 2 (SARS-CoV-2) status. In Tanzania, all clinical research was suspended at the onset of the pandemic, there was unavailability of personal protective equipment, and the lack of well-equipped laboratories and unavailability of some reagents prevented conclusive diagnosis of SARS-CoV-2. In Rwanda, COVID-19 primarily affected case enrollment, presumably because of a significant reduction of hospital admissions and hence fewer eligible cases. Furthermore, most clinical activities and efforts of physicians were oriented around COVID-19-related activities.

### Lessons Learned for Successful Implementation of MITS

All 4 sites learned the following lessons: All sites determined that understanding the opinions of stakeholders and the community is imperative when implementing a new intervention. Sites found it advantageous to share the research protocol with stakeholders and healthcare workers and to inform them of the MITS process. The autonomy of family and relatives of the deceased needs to be fully respected and prioritized. Sites found it advantageous to offer grief counseling to the family and relatives before the consenting process and allow sufficient time for grief expression. As observed in other MITS studies, the Nepalese team found that the counseling and consent process was made more complex when many family members were involved; the team found it helpful to identify 1 or 2 key family members to work with as part of the counseling and consent process [14].

Sites identified specific strategies to alleviate concerns and increase acceptability among families and clinicians. Initially the Nepalese clinicians involved in patient care were skeptical of MITS outcomes, but when the MITS findings were periodically shared with them, they gradually understood the importance of the procedure, and it became well accepted. Engaging clinicians involved in patient care in the process for determining of cause of death also facilitated their support and acceptance of MITS. In Rwanda, the procedure became acceptable by relatives after engaging a well-trained psychological support team. Once MITS was understood and the value demonstrated, clinicians and nursing staff had positive attitudes and were motivated to support the study.

The inadequacies of the infrastructure and basic instruments and materials required for MITS were overcome by upgrading facilities and procuring needed materials. All sites felt that when researchers are considering the feasibility of implementing MITS, autopsy and facility resources should be assessed and the potential to improve facilities taken into consideration. Furthermore, when testing and analysis that is beyond the existing capacity of a new study site is required, collaboration with national laboratories should be considered. In Nepal, the need for additional laboratory tests was evaluated on a case-by-case basis after reviewing initial results for establishment of cause of death and when necessary, the study staff collaborated with national laboratories to perform specific tests, for example, GeneXpert for tuberculosis.

The lack of adequate clinical records was a constraint at all the sites. In Nepal, it was partially overcome by recording the contact details of the consenting family and relatives for follow-up queries and communication of results. This was particularly helpful in cases where MITS results identified conditions or diseases that could potentially affect the health of other family members. This helped to gather further clinical information that was not available during the MITS procedure and also benefited the family members as they were informed of the conditions of their loved ones. In compliance with national law, at CHUB in Rwanda, the team designed and implemented a MITS request form completed by the treating clinician and included important antemortem clinical and diagnostic information. This required revisiting, recollecting, and validating data from the hospital file of each case.

The successful implementation of MITS in these 4 sites demonstrates the feasibility of implementing MITS in limited-resource settings and highlights that MITS can contribute valuable information on cause of death. MITS remains an important alternative tool to CDA to support cause of death determination, particularly in settings with limited access to antemortem diagnostic testing.

## CONCLUSION

All 4 study sites were successful in their implementation of MITS. Despite geographical and cultural differences, all 4 sites had to overcome similar challenges in implementing MITS. Documenting the challenges and sharing the lessons learned across different settings and contexts builds on the limited existing body of knowledge and can be used in planning future MITS studies across a growing number of populations and settings, particularly in LMICs.

## Notes

***Acknowledgments.*** We would like to acknowledge the members of the MITS Surveillance Alliance and trainers and facilitators involved in capacity-building programs. We thank the research team of each of the study sites and the staff and management of the hospitals for their support in accomplishing the projects.

***Supplement sponsorship.*** This supplement is sponsored by MITS Surveillance Alliance Secretariat, led by RTI International, with funding from the Bill and Melinda Gates Foundation.

***Financial support***. This work was supported by the MITS Surveillance Alliance. This work was supported, in whole or in part, by the Bill & Melinda Gates Foundation (grant number OPP1180554). Under the grant conditions of the Foundation, a Creative Commons Attribution 4.0 Generic License has already been assigned to the Author Accepted Manuscript version that might arise from this submission. MITS Surveillance Alliance provided payment and study materials (MITS Kits) to Gandaki Medical College, Kilimanjaro Christian Medical Centre, Moshi, Tanzania, Kigali University Teaching Hospital/CHUK, Kigali, Rwanda, and University Teaching Hospital Butare/CHUB, Huye, Rwanda.

***Potential conflicts of interest.*** The authors: No reported conflicts of interest. All authors have submitted the ICMJE Form for Disclosure of Potential Conflicts of Interest. Conflicts that the editors consider relevant to the content of the manuscript have been disclosed.
